# Add-on Pegylated Interferon Alpha-2a Therapy in Chronic Hepatitis B Japanese Patients Treated with Entecavir

**DOI:** 10.1155/2017/2093847

**Published:** 2017-04-11

**Authors:** Hideyuki Tamai, Yoshiyuki Ida, Naoki Shingaki, Ryo Shimizu, Kazuhiro Fukatsu, Masahiro Itonaga, Takeichi Yoshida, Yoshimasa Maeda, Kosaku Moribata, Takao Maekita, Mikitaka Iguchi, Jun Kato, Masayuki Kitano

**Affiliations:** Second Department of Internal Medicine, Wakayama Medical University, 811-1 Kimiidera, Wakayama City, Wakayama 641-0012, Japan

## Abstract

Entecavir requires long-term administration. Pegylated interferon (PEG-IFN) therapy leads to significant reduction of hepatitis B surface antigen (HBs Ag) levels. This study aimed to assess the safety and efficacy of adding PEG-IFN-*α*-2a to entecavir toward cessation of entecavir. A total of 23 patients treated with entecavir underwent add-on PEG-IFN-*α*-2a therapy (90 *μ*g per week) for 48 weeks. Viral response (VR) was defined as more than 50% reduction of baseline hepatitis B surface antigen (HBs Ag) level at 72 weeks from the start of therapy. Complete response (CR) was defined as the decline of HBs Ag levels <100 IU/mL. Hepatitis B e antigen (HBe Ag) seroconversion rate was 25% (2/8), and VR rate was 52% (12/23). CR was observed in four patients (17%). However, CR rate in baseline HBs Ag level <2000 IU/mL and HBe Ag negative patients was 50% (4/8). Univariate analysis showed that the percentage of HBs Ag level reduction at week 12 was significantly associated with VR. The area under the curve value was 0.848. Adding PEG-IFN-*α*-2a to entecavir has limited efficacy. The percentage reduction of HBs Ag level at week 12 may be a useful predictor for VR.

## 1. Introduction

The current standard treatment for patients with chronic hepatitis B (HB) is pegylated interferon alpha-2a (PEG-IFN-*α*-2a) or nucleoside analogs (NAs). NAs selectively inhibit viral DNA replication and are extremely safe and effective. In Japan, entecavir has been approved by the national health insurance in 2006. At present, it is the most widely used first-line NA because of its low risk of resistant viruses compared with lamivudine [1]. However, because relapse risk of severe hepatitis after discontinuation is high, long-term administration is needed [2–4]. Furthermore, there are some notable problems associated with long-term administration of entecavir such as increased risk of resistant viruses, safety concerns, teratogenicity, and high medical costs. In contrast, although the antiviral effect of interferon (IFN) is inferior to that of NAs, IFN activates antiviral immunity and can lower hepatitis B surface antigen (HBs Ag) level compared with entecavir [5].

In 2001, Serfaty et al. first reported that sequential therapy with lamivudine and IFN-*α* can induce a sustained virologic response, including HBs seroconversion, in patients with chronic hepatitis B not responding to IFN-alpha alone [6]. Chen et al. reported that long-term combination therapy with IFN and NA achieved a high rate of HBs Ag clearance [7]. Notably, Kittner et al. demonstrated that add-on PEG-IFN-*α*-2a induced HBsAg seroconversion in 2 out of 12 patients [8]. In Japanese guidelines, risk assessment of relapse following cessation of NAs has been shown, and NAs may be safely stopped in patients whose HBsAg level decreased to <1.9 Log IU/mL [9]. PEG-IFN therapy leads to notable reduction of HBs Ag levels, whereas the levels did not decrease in patients treated with entecavir alone [10]. Therefore, if a marked reduction of the HBs Ag level appears with additional IFN therapy in patients being treated with entecavir, entecavir may be discontinued safely. To date, there is insufficient evidence of how much HBs Ag level decreases in Japanese patients with chronic hepatitis B and how many patients have achieved cessation of entecavir with add-on IFN. Therefore, in this study, we aimed to assess the safety and efficacy of adding PEG-IFN-*α*-2a to entecavir.

## 2. Patients and Methods

### 2.1. Patients

A total of 23 patients treated with entecavir underwent add-on PEG-IFN-*α*-2a therapy in our hospital between July 2012 and May 2014. Patients were enrolled if all of the following criteria were fulfilled: (1) HBs Ag level > 100 IU/mL; (2) 20 years of age or older; (3) granulocyte count > 1500/mm^3^; (4) platelet count > 70,000/mm^3^; and (5) hemoglobin (Hb) levels > 10 g/dL. The exclusion criteria were (1) hepatic failure or cancer; (2) using* shosaikoto* (a traditional Chinese medicine); (3) intractable heart disease; and (4) uncontrollable psychoneurotic disorders. All enrolled patients underwent abdominal ultrasonography and contrast computed tomography for diagnosis of liver cirrhosis and hepatocellular carcinoma (HCC) screening within 1 month before the start of therapy. Liver cirrhosis was clinically diagnosed using the morphologic appearance with portal hypertension, such as portosystemic shunt or hypersplenism on imaging or liver histology.

This is a prospective cohort study of add-on PEG-IFN-*α*-2a therapy in Japanese patients with chronic hepatitis B treated with entecavir. All study protocols were approved by the ethics committee of our hospital (number 1092). Written informed consent was obtained from all patients included in this study. This study was registered on the University Hospital Medical Information Network (trial ID 8693).

### 2.2. Treatment Regimens

The standard dose of PEG-IFN-*α*-2a (Pegasys®; Roche, Basel, Switzerland) in Japan was used; 90 *μ*g PEG-IFN-*α*-2a was administered subcutaneously once a week for 48 weeks [5]. A dose of 0.5 mg entecavir (Baraclude; Bristol-Myers Squibb, New York, USA) was administered daily even after the introduction of add-on PEG-IFN-*α*-2a treatment. PEG-IFN-*α*-2a was interrupted based on the following criteria: (1) Hb < 8.5 g/dL; (2) granulocyte count < 500/mm^3^, or the platelet count < 25,000/mm^3^; and (3) attending physician deeming necessary due to adverse events. The treatment could be restarted if cytopenia improved. If there was no improvement in hematological parameters or adverse events within four weeks, this therapy was discontinued.

### 2.3. Laboratory Tests

Hepatitis B virus (HBV) genotype was determined using enzyme immunoassay. Hepatitis B e antigen (HBe Ag) level was measured using chemiluminescent immunoassay. HBs Ag levels were measured using the Archect HBs-QT (Abbott Laboratories, Chicago, IL, USA). HBV-DNA was measured using the COBAS TaqMan PCR assay (Roche Diagnostics, Branchburg, NJ, USA). Single nucleotide polymorphism (SNP) of interleukin-28B (IL-28B) host genotype (rs8099917) [11] was also evaluated after obtaining written informed consent for genome analysis from each patient. Homozygosity for the major allele (T/T) was defined as IL-28B major type, and heterozygosity (T/G) or homozygosity for the minor allele (G/G) as IL-28B minor type. In addition to biochemical analyses including serum alanine aminotransferase (ALT), *γ*-glutamyl transpeptidase (*γ*GTP), total bilirubin, prothrombin time, and albumin, levels of fibrosis markers (Type IV collagen 7S and hyaluronic acid) and alpha-fetoprotein (AFP) were measured within one month before the start of therapy. During therapy, blood cell counts were performed every week before treatment up to 8 weeks after the start of therapy, and HBs Ag, HBs antibody, HBe Ag, HBe antibody, and HBV-DNA levels and biochemical analyses (liver and renal functional tests) were measured every 4 weeks up to 24 weeks after the end of therapy.

### 2.4. Assessment of Effectiveness and Safety

The final outcome was evaluated at 24 weeks from the end of add-on PEG-IFN-*α*-2a therapy. For the analysis of factors contributing to the HBs Ag reduction, viral response (VR) was defined as more than 50% reduction of the baseline HBs Ag level. In Japanese guidelines, 1.9 log IU/mL for HBs Ag is set as the cutoff level at the time of stopping NAs in patients with negative results for HBe Ag and HBV-DNA < 3.0 log IU/mL [9]. Accordingly, complete response (CR) was defined as achievement of HBs Ag level <100 IU/mL.

Patients were assessed for safety and tolerability during treatment by their attending physician who monitored adverse events and laboratory abnormalities, such as blood cell counts, every week up to week eight. Thereafter, the patients were followed up monthly to 24 weeks after the end of therapy. The incidence and reasons for therapy discontinuation were analyzed.

### 2.5. Statistical Analysis

Therapeutic effectiveness was determined using an intention-to-treat (ITT) analysis that included patients who did not complete the scheduled course of therapy. Predictive factors for VR were also analyzed using a ITT analysis. Mann–Whitney* U* test was used to analyze continuous variables. Fisher's exact test or the chi-square test was used to analyze categorical variables. Each optimal cutoff value for continuous variables of VR-predicting factors was decided by the Youden Index method on the basis of the receiver operating characteristics (ROC) curve. The VR predictability of significant contributing factors was evaluated by measuring the area under the curve (AUC). The sensitivity, specificity, positive predictive value (PPV), negative predictive value (NPV), and accuracy for VR were calculated. Values of *p* < 0.05 were considered significant. The statistical software used was SPSS version 20.0J for Windows (SPSS, Inc., Tokyo, Japan).

## 3. Results

### 3.1. Baseline Background Factors

The patients' baseline characteristics are summarized in Table 1.

### 3.2. Therapeutic Effectiveness

**Figure 1:**
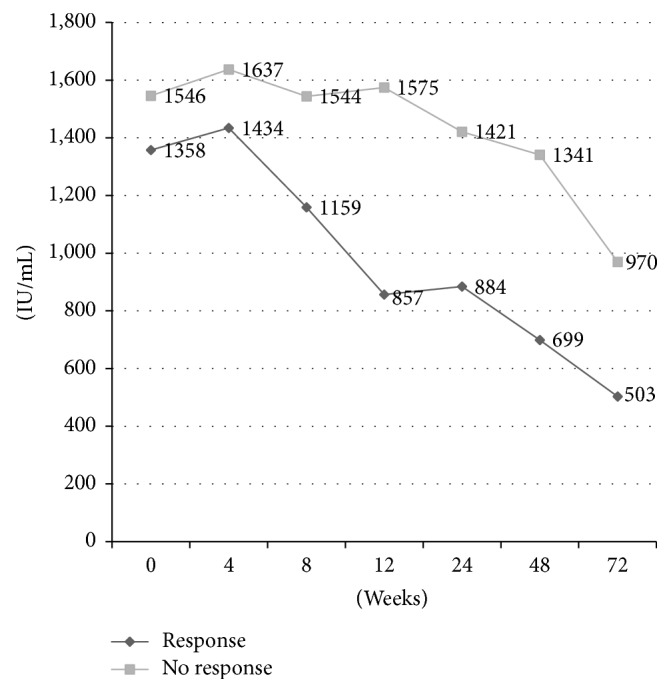
Comparison of HBs Ag levels between viral response and no viral response groups. Data were expressed as medians.

**Figure 2:**
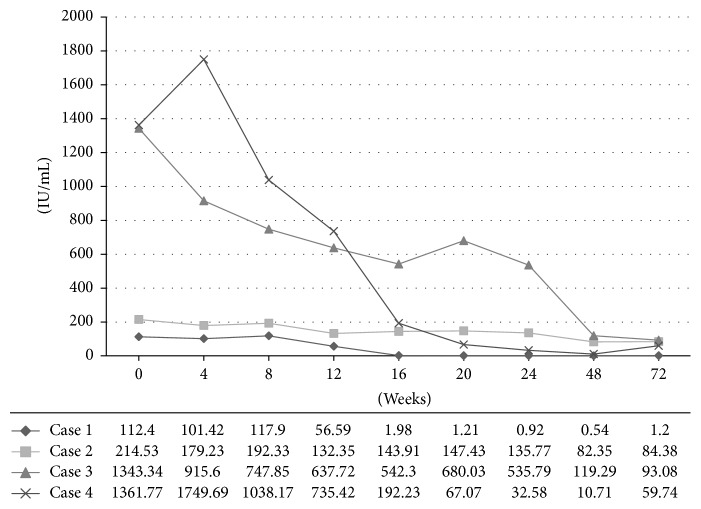
Changes of HBs Ag levels of patients with complete response.

HBe seroconversion was seen in 2 patients (25%). Although no HBs Ag disappearance was seen, CR was observed in 4 patients (17%), and all of them were HBe Ag negative patients. CR rate in HBe Ag negative patients was 27% (4/15). VR rate was 52% (12/23). Comparison of HBs Ag levels between VR and no VR groups is shown in Figure 1. Changes of HBs Ag levels of patients with complete response are shown in Figure 2. CR rate in baseline HBs Ag level <2000 IU/mL and HBe Ag negative patients was 50% (4/8).

There were no patients with new-onset hepatocellular carcinoma during the observation period. Entecavir could be discontinued in one patient by seroclearance of HBs Ag after one year from the end of add-on PEG-IFN therapy.

### 3.3. Contributing Factors for VR and Prediction of VR

Comparisons of pre- and on-treatment factors between the VR and no VR groups are shown in Table 2. ALT level was the only significant pretreatment factor associated with VR. Age, sex, time of previous entecavir therapy, HBe Ag positivity, HBV genotype, HBs Ag level, and interleukin-28B polymorphism were not significant. Among on-treatment factors, the percentage reductions of HBs Ag levels at weeks 12 and 16 were significant contributing factors for VR. AUC values according to significant contributing factors for VR are shown in Table 3. The AUC value of the percentage reduction of HBs Ag at week 12 was the highest. The sensitivity, specificity, PPV, NPV, and accuracy for VR are summarized according to significant contributing factors shown in Table 4. Predictability of VR using percentage reduction of HBs Ag level at week 12 was equal to that at week 16.

### 3.4. Safety and Tolerability

The overall adverse events are shown in Table 5. A 38-year-old female discontinued PEG-IFN therapy due to arrhythmia at 36 weeks after the start of therapy. She neither was advanced in age nor had cirrhosis. Treatment discontinuation rate was 4% (1/23). Treatment interruption rate was 17% (4/23).

## 4. Discussion

This is the first report of add-on PEG-IFN-*α*-2a therapy in Japanese patients treated with entecavir. Liaw et al. reported that a dose of 180 *μ*g PEG-IFN-*α*-2a was efficacious and beneficial for patients infected with HBV genotype B or C compared with a dose of 90 *μ*g dose [12]. In addition, the dosage of 180 *μ*g PEG-IFN-*α*-2a was used in combination with NA in many countries [13–15]. In the present study, however, a dosage of 90 *μ*g PEG-IFN-*α*-2a was used because it was recommended by a Japanese clinical trial of PEG-IFN-*α*-2a monotherapy for patients with chronic active hepatitis B [5]. Additionally, this dosage was considered to be safer compared with a 180 *μ*g dose, which is also the recommended dose for cirrhotic patients infected with hepatitis C virus in Japan [16]. The discontinuation rate due to adverse effects in the present study was low (4%). Also, despite the fact that 11 cirrhotic patients were included in the present study, there was no discontinuation in cirrhotic patients. Therefore, this regimen was considered to be safe. As advanced fibrosis is common in older patients with HBe Ag negative chronic hepatitis, and NAs are the first-line treatment for cirrhotic patients, the proportion of aged and cirrhotic patients who are receiving long-term entecavir therapy is relatively high compared with that of patients with HBe Ag positive chronic hepatitis. Therefore, the lower dosage of 90 *μ*g of PEG-IFN-*α*-2a for add-on therapy would be a safe and more assessable dose even for aged and/or cirrhotic patients receiving long-term entecavir therapy.

The CR rate in the present study was low (17%), and the disappearance of HBs Ag or HBs seroconversion was not seen. If the aim of add-on PEG-IFN-*α*-2a therapy was to safely discontinue entecavir, this regimen would be considered insufficient. Regarding the efficacy of adding PEG-IFN to NA therapy, Ouzan et al. reported that the addition of 180 *μ*g of PEG-IFN-*α*-2a for a maximum of 96 weeks based on HBsAg-titer monitoring led to a loss of HBsAg and cessation of NA therapy in six out of ten patients (60%), with no relapse [17]. Marcellin et al. reported that the rates of HBs Ag loss were significantly higher in the group treated with tenofovir plus PEG-IFN for 48 weeks than in the group (6.5%) treated with tenofovir plus PEG-IFN for 16 weeks and tenofovir for 32 weeks (0.5%) [18]. Therefore, longer-term add-on PEG-IFN-*α*-2a therapy would be needed for the cessation of NA. However, adverse effects are common in high-dose IFN therapy especially in cirrhotic patients and the elderly. Adequate doses of PEG-IFN for long-term treatment should be clarified in the future.

In the present study, HBs Ag levels in most of the patients were decreased in varying degrees during add-on PEG-IFN-*α*-2a, and the decrease continued even after add-on PEG-IFN-*α*-2a therapy. Furthermore, this reducing effect of HBs Ag level gradually appeared 12 weeks after the start of add-on PEG-IFN-*α*-2a. This phenomenon is significant to the study because it is not seen in NA therapy. It was reported that high HBs Ag level is a risk factor for carcinogenesis in inactive HBV carriers with negative HBe Ag and low HBV-DNA level [19]. As most of the patients in the present study have shown similar condition with inactive low HBV-DNA carrier by long-term entecavir therapy, the reduction effect of HBs Ag by adding PEG-IFN-*α*-2a on entecavir may lower carcinogenic risk. To confirm this, further study is needed.

Recent multicenter randomized trial of 180 *μ*g of PEG-IFN-*α*-2a with entecavir for patients with HBe Ag positive chronic hepatitis B for 48 weeks showed that the rate of HBe Ag loss at 72 weeks was 32%, which was higher than the 18% in entecavir monotherapy [20]. However, no HBs Ag seroconversion was seen at 72 weeks even in add-on therapy. Although the number of HBe Ag positive patients in the present study was small, the rate of HBe Ag loss by add-on therapy was 25%, with no HBs Ag loss or seroconversion seen in HBe Ag positive patients. Accordingly, it might be difficult that add-on PEG-IFN-*α*-2a for HBe Ag positive patients treated with entecavir achieves HBs Ag disappearance. In contrast, there are no reports of HBs Ag loss or seroconversion due to the addition of PEG-IFN-*α*-2a to entecavir for HBe Ag negative patients. In the present study, CR rate in HBe Ag negative patients was 27%. Furthermore, in the case of patients with HBe Ag negative and baseline HBs Ag level <2000 IU/mL, the CR rate rose to 50%. Therefore, it is conceivable that optimal clinical indication of add-on PEG-IFN-*α*-2a therapy for patients treated with entecavir is considered as patients with HBe Ag negative and low HBs Ag level (at least <2000 IU/mL).

Baseline ALT level and the reduction of HBs Ag level were significant contributing factors for VR (more than 50% reduction of baseline HBsAg level). Buster et al. reported that high levels of baseline ALT were among the predictive factors of viral response in PEG-IFN monotherapy, and genotypes B and C patients who had high levels of baseline ALT and low levels of HBV-DNA were good candidates for IFN monotherapy [21]. However, predicting VR using baseline ALT level was inferior compared with using the reduction of HBs Ag level. At present, there is no established method for predicting treatment response of PEG-IFN using pretreatment factors except for genotype A [16]. Notably, some reports indicated that HBs Ag level or the reduction of HBs Ag at week 12 was a strong predictor of virological response in peg-IFN monotherapy [22–25]. It was also reported that titers of baseline HBs Ag and HB core-related Ag were useful predictors of response to PEG-IFN [26, 27]. In our study, there were no significant differences in baseline HBs Ag levels between VR and no VR groups. As the predictability of VR using the reduction of HBs Ag level at week 12 was equal to that at week 16, the optimal time point to predict VR would be 12 weeks after the start of therapy.

Certain limitations must be considered when interpreting the results of the present study. First, this represents a single-center, preliminary, small-scale, prospective cohort study without no control group. Although the effect of the reduction of HBs Ag by entecavir alone is small [28], it should be clear whether the reduction of HBs Ag was due to the add-on PEG-IFN therapy or due to elongation of entecavir. Additionally, the number of the patients in our study is too small to draw a conclusion. A large scale randomized controlled trial is needed to validate our findings. Second, we defined VR as more than 50% reduction of baseline HBs Ag level to evaluate the response of adding PEG-IFN, for convenience, and conclusion cannot be drawn as to whether this definition was appropriate. Because the range of HBs Ag levels in patients with HBV is very wide, and the meaning of a 50% reduction of HBs Ag in clinical practice is unknown. Generally, efficacy of antiviral therapy for patients with chronic hepatitis B is evaluated by ALT or HBV-DNA levels. However, as ALT and HBV-DNA levels of the patients being treated with entecavir are very low, these markers are not available to evaluate outcomes of add-on PEG-IFN to NAs. It is reported that HBs Ag quantification is useful for monitoring natural history and treatment outcomes [29]. As HBs Ag seroclearance is required at the time of stopping NA as stated in international guidelines [30], CR should be defined as HBs Ag seroclearance in add-on PEG-IFN therapy to NAs. Third, this study is a report of Japanese patients mainly infected with genotype C HBV. As IFN has varying response, our results cannot be applied to other genotypes or races. Fourth, patients with previous lamivudine failure or breakthrough hepatitis during entecavir treatment were not included in the present study. Therefore, efficacy add-on PEG-IFN therapy for mutant virus to NAs could not be evaluated.

In conclusion, adding PEG-IFN-*α*-2a to entecavir is safe but has limited efficacy. The percentage reduction of HBs Ag level at week 12 may be a useful predictor for VR. The best candidates by add-on PEG-IFN for the discontinuation of entecavir would be those who are HBe Ag negative and with low HBsAg level (<2000 IU/mL).

## Figures and Tables

**Table 1:** Patients' baseline characteristics (*n* = 23).

Age (years) (range)	47 (30–65)
Body weight (kg)	64.5 (45.0–110.5)
Body mass index (kg/m^2^)	23.2 (17.9–32.3)
Prior interferon therapy	7 (30%)
History of HCC treatment	6 (26%)
Genotype B/C	2 (9%)/21 (91%)
Duration of prior entecavir (days)	1379 (371–2410)
HBe Ag positive	8 (35%)
HBV-DNA (log IU/mL)	2.1 (0–2.4)
HBs Ag (IU/mL)	1361.77 (112.40–19673.70)
Liver cirrhosis (%)	11 (48)
IL28B (rs8099917) major	20 (87)
White blood cell (/mm^3^)	4870 (2920–9100)
Hemoglobin (g/dL)	14.8 (12.0–16.5)
Platelets (×10^4^/mm^3^)	17.8 (9.3–28.4)
ALT (IU/L)	21 (10–47)
*γ*GTP (IU/L)	24 (11–82)
Alpha-fetoprotein (ng/mL)	2.2 (1–6.9)
Type IV collagen 7S (ng/mL)	3.8 (2.5–5.4)
Hyaluronic acid (ng/mL)	48 (10–266)

HCC, hepatocellular carcinoma; HBe Ag, hepatitis B e antigen, HBs Ag, hepatitis B surface antigen; HBV, hepatitis B virus; IL, interleukin; ALT, alanine aminotransferase; *γ*GTP, *γ*-glutamyltransferase.

Values are expressed as medians (range) or numbers of patients (percent).

**Table 2:** Comparison of pre- and on-treatment factors between the viral response and no viral response groups.

Factors	VR (*n* = 12)	No VR (*n* = 11)	*p*
Age (years) (range)	52 (36–62)	44 (30–65)	0.211
Sex (male/female)	10/2	6/5	0.193
Body weight (kg)	65.9 (53.0–91.0)	56.6 (45–110.5)	0.104
BMI (kg/m^2^)	23.9 (17.9–30.1)	21.2 (17.9–32.3)	0.151
Prior interferon therapy	2	5	0.193
History of HCC treatment	4	2	0.640
Genotype B/C	1/11	1/10	1.000
Duration of prior entecavir (days)	1831 (560–2275)	1316 (371–2410)	0.190
HBe Ag positive	4	4	1.000
HBV-DNA (log IU/mL)	2.1 (0–2.1)	2.1 (0–2.4)	0.740
HBs Ag (IU/mL)	1358 (112–5807)	1546 (778–19674)	0.651
Liver cirrhosis	7	4	0.414
IL28B (rs8099917) major	11	9	0.590
White blood cell (/mm^3^)	4765 (3260–9100)	5230 (2920–6630)	0.740
Hemoglobin (g/dL)	14.9 (14.0–15.8)	14.6 (12.0–16.5)	0.379
Platelets (×10^4^/mm^3^)	18.8 (9.3–28.4)	17.1 (10.4–25.2)	0.487
AST (IU/L)	25 (14–34)	23 (14–31)	0.449
ALT (IU/L)	24 (16–47)	17 (10–32)	0.032
*γ*GTP (IU/L)	29 (13–81)	22 (11–82)	0.288
AFP (ng/mL)	2.2 (1.0–6.9)	2.7 (1.2–4.6)	0.651
Type IV collagen 7S (ng/mL)	3.8 (2.5–5.4)	3.5 (2.7–4.7)	0.833
Hyaluronic acid (ng/mL)	62 (10–266)	44 (26–94)	0.379
HBs Ag at week 4 (IU/mL)	1434 (101–5525)	1637 (694–21699)	0.651
Percentage reduction of HBs Ag at week 4	6 (−35–32)	−1 (−12–21)	0.880
HBs Ag at week 8 (IU/mL)	1159 (118–4286)	1544 (608–19397)	0.288
Percentage reduction of HBs Ag at week 8	22 (−5–51)	1 (−37–38)	0.091
HBs Ag at week 12 (IU/mL)	857 (57–4391)	1575 (591–23678)	0.134
Percentage reduction of HBs Ag at week 12	35 (−4–58)	9 (−29–41)	0.004
HBs Ag at week 16 (IU/mL)	966 (2–3703)	1329 (570–27476)	0.190
Percentage reduction of HBs Ag at week 16	32 (13–98)	14 (−40–36)	0.007

VR, viral response; HCC, hepatocellular carcinoma; HBe Ag, hepatitis B e antigen, HBs Ag, hepatitis B surface antigen; HBV, hepatitis B virus; IL, interleukin; ALT, alanine aminotransferase; *γ*GTP, *γ*-glutamyltransferase.

Values are expressed as medians (range) or numbers of patients (percent).

**Table 3:** Areas under the curve according to significant contributing factors for viral response.

Factors	AUC	*p*	95% CI
ALT	0.761	0.034	0.561–0.961
Percentage reduction of HBs Ag at week 12	0.848	0.005	0.687–1.000
Percentage reduction of HBs Ag at week 16	0.833	0.007	0.687–1.000

AUC, area under the curve; CI, confidential interval; ALT, alanine aminotransferase; HBs Ag, hepatitis B antigen.

**Table 4:** Predictability of viral response.

Factors	Sensitivity	Specificity	PPV	NPV	Accuracy
ALT (>20 IU/L)	67% (8/12)	64% (7/11)	67% (8/12)	64% (7/11)	65% (15/23)
Reduction rate of HBs Ag at week 12 (>25%)	75% (9/12)	82% (9/11)	82% (9/11)	75% (9/12)	78% (18/23)
Reduction rate of HBs Ag at week 16 (>30%)	75% (9/12)	82% (9/11)	82% (9/11)	75% (9/12)	78% (18/23)

PPV, positive predictive value; NPV, negative predictive value; ALT, alanine aminotransferase; HBs Ag, hepatitis B antigen.

**Table 5:** Adverse events during treatment.

	Number
Treatment discontinuation due to adverse event (total)	1
Arrhythmia	1
Treatment interruption due to adverse event (total)	4
Acute pyelonephritis	1
Acute pharyngitis	1
Thrombocytopenia	1
Vertigo	1
Other adverse events (total)	10
Dermatitis	4
Depression	3
Fever	2
Ischemic colitis	1
